# Supplementary material for: Intrinsic attraction driving the high temperature performance of additively manufactured aluminum alloys
Source: Nat Commun. 2026 Apr 7;17:4931. doi: 10.1038/s41467-026-71390-3 (PMC13234029; doi:10.1038/s41467-026-71390-3)
Supplement: Supplementary file 1 — Supplementary Information [file 41467_2026_71390_MOESM1_ESM.pdf]

Supplementary Information for

Intrinsic attraction driving the high temperature performance of additively manufactured aluminum alloys

*Yueting Wang[1], Chengzhe Yu[1], Kefu Gan[2, *], Tiechui Yuan[1], Ruidi Li[1,3, *]*

[1] *State Key Laboratory of Powder Metallurgy, Central South University, Changsha 410083, China*

[2] *School of Materials Science and Engineering, Central South University, Changsha 410083, China*

[3] *National Key Laboratory of Science and Technology on High-Strength Structural Materials, Central South University, Changsha, 410083, China*

*Corresponding author.

Email: liruidi@csu.edu.cn (R. Li), gankefu@csu.edu.cn (K. Gan)

The file includes:

Supplementary Figs. 1 to 16

Supplementary Tables 1 to 3

Supplementary Notes :1 to 7

Supplementary References 1-14

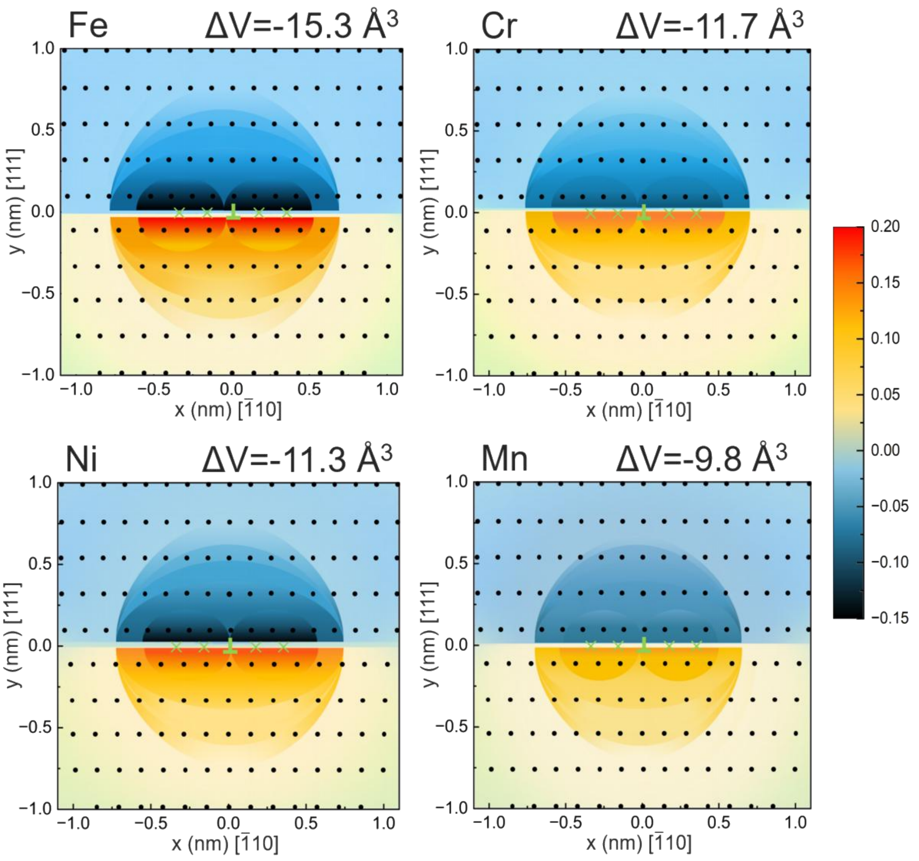

Supplementary Fig. 1 The plot of the solute-dislocation interaction energy U (x_i, y_j) for all the solute locations (x_i, y_j) in and around the core for Fe, Ni, Cr and Mn solutes. The orange (positive energy) corresponds to expansion, while the blue (negative energy) corresponds to binding. The interaction energy was calculated using first-principles methods; the crosses indicate the Burgers vector.

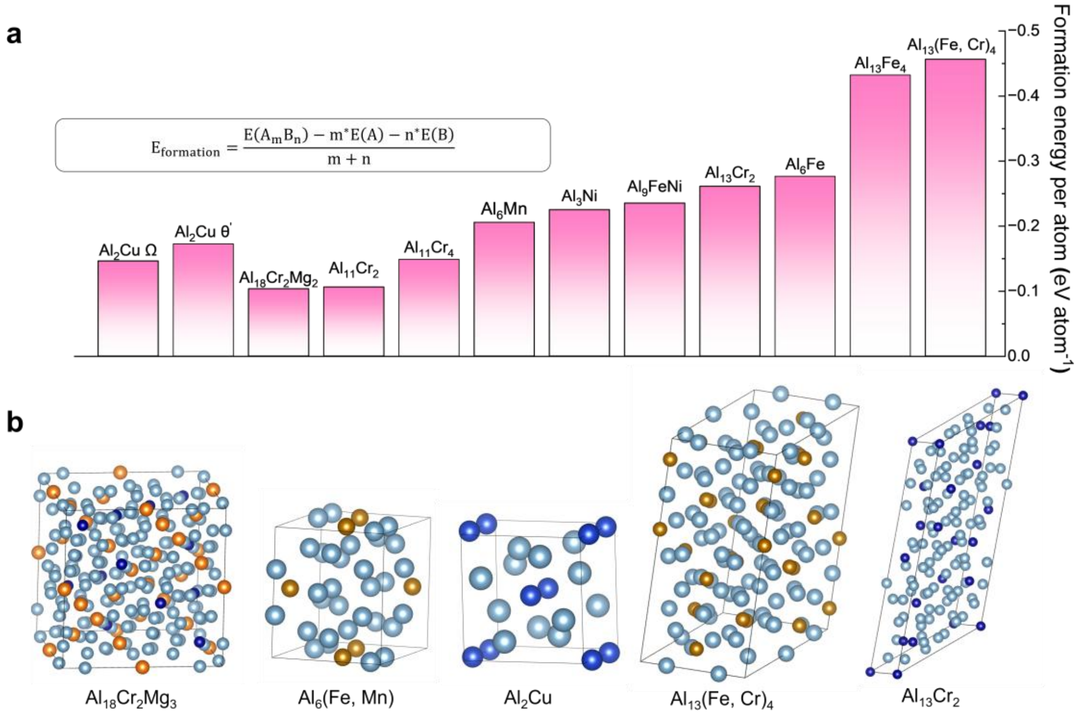

Supplementary Fig. 2 The DFT simulation results compare the formation energy of each atom of the Al_xM_y precipitates, where the greater the negative value indicates the stronger the atomic bond. (a) The higher the formation energy, the better the high-temperature stability of the precipitates. (b) Schematic diagram of the crystal structure of some precipitates.

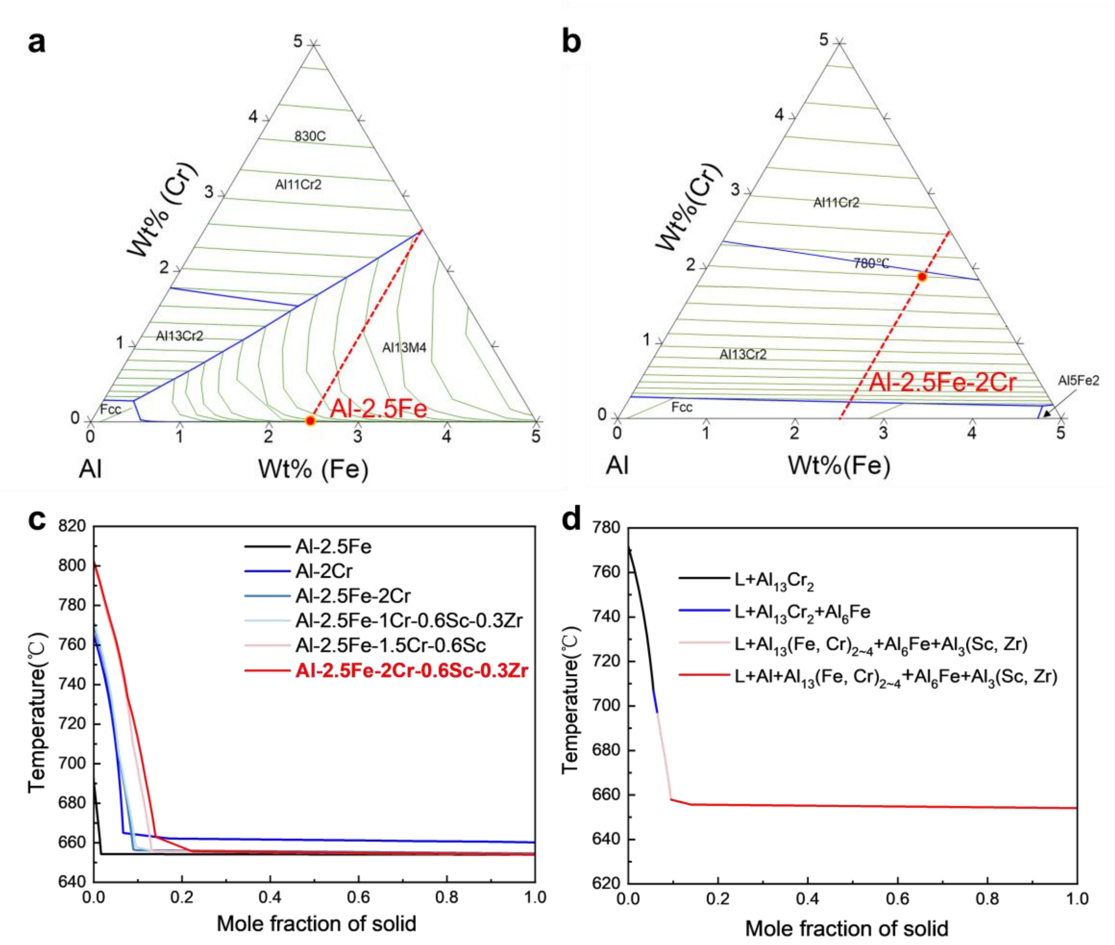

Supplementary Fig. 3 The optimal Fe and Cr content based on thermodynamic calculation: (a) calculated equilibrium liquidus projection, and (b) calculated non-equilibrium liquidus projection (excluding θ-Al13Fe4 phase) in the Al-Fe-Cr ternary system. Scheil sequences of (c) Al-2.5Fe, Al-2Cr, Al–2.5Fe-xCr-0.6Sc-0.3Zr (x=1, 1.5 and 2 wt.%) alloys under non-equilibrium solidification conditions and (d) 2.5Fe-2Cr-0.6Sc-0.3Zr (wt. %) alloy under non-equilibrium solidification condition.

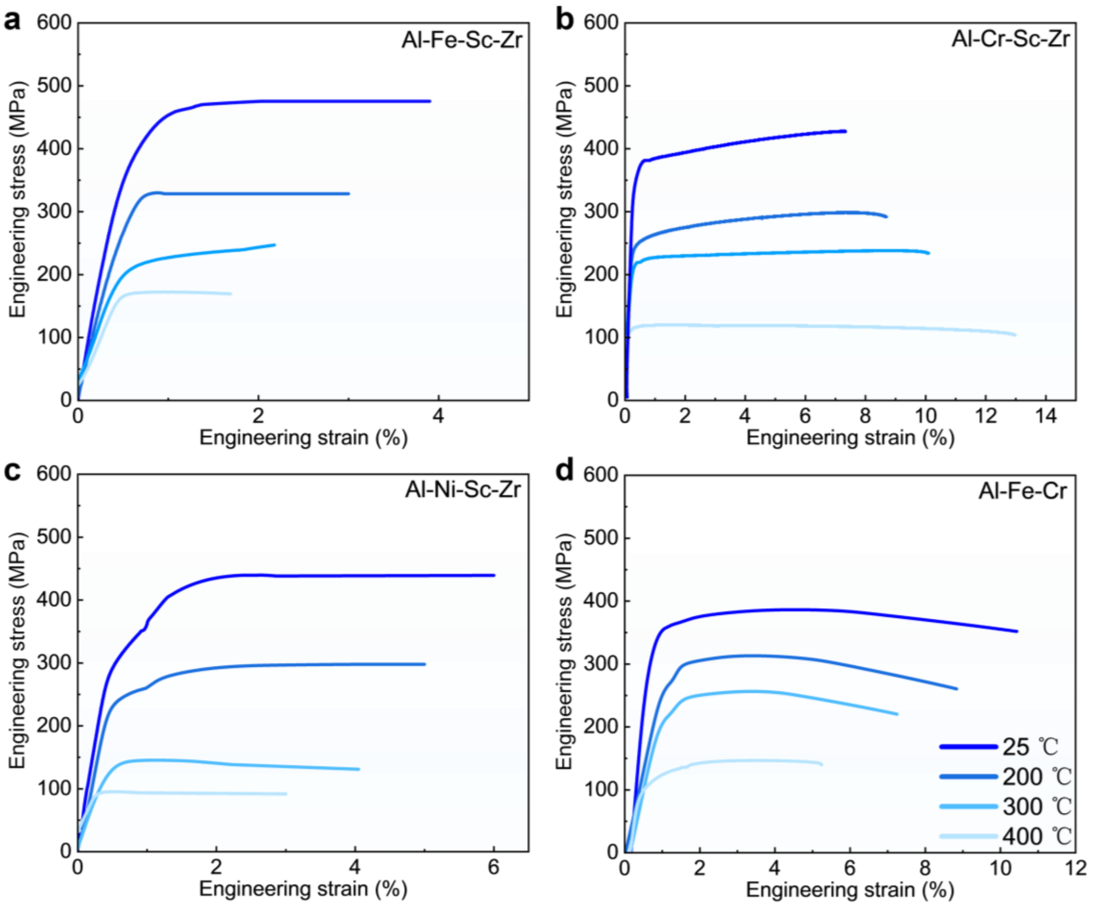

Supplementary Fig. 4 Mechanical properties of heat-resistant aluminum alloys with different compositions. (a~d) Representative tensile data of Al-Fe-Sc-Zr, Al-Cr-Sc-Zr, Al-Ni-Sc-Zr and Al-Fe-Cr after heat treated conditions (consistent with IA alloy at 325 °C for 4h).

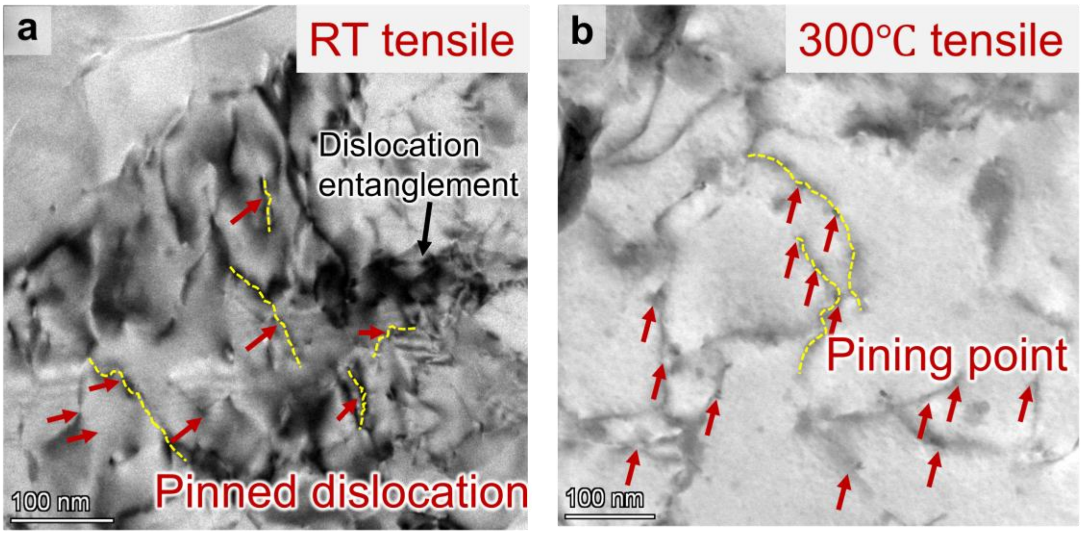

Supplementary Fig. 5 Dislocations pinned can be observed in both (a) room temperature and (b) high temperature tensile samples.

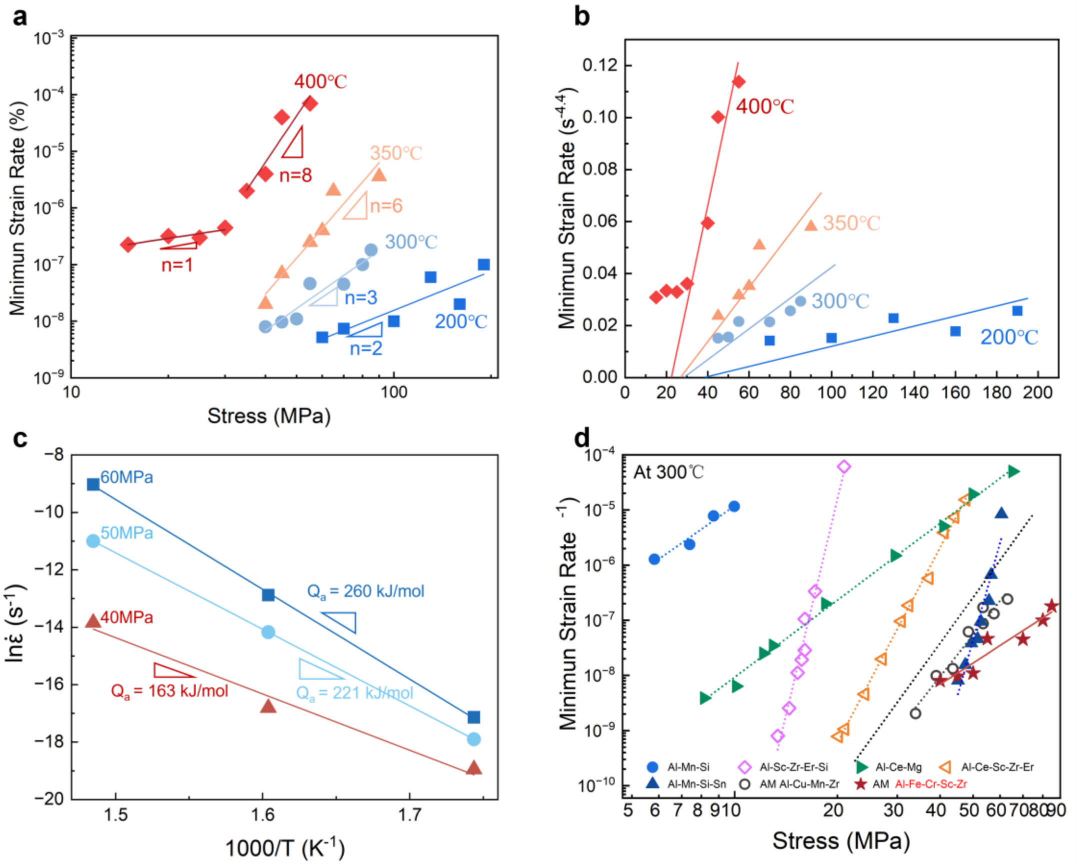

Supplementary Fig. 6 (a) Double-logarithmic plots of the minimum strain rate with stress for IA alloy at 200, 300, 350 and 400 ℃. (b) The creep threshold stress of IA alloy at each test temperature (n = 4.4 for pure Al). (c) Creep activation energies measured at 40, 50 and 60 MPa by Arrhenius formula. The slopes are equal to -$Q_{app}/R_{g.}$ (d) Creep behavior of the AM IA alloy compared with other heat-resistant Al alloys from the literature: Al-Mn-Si[1], Al-Mn-Si-Sn[1],, Al-Sc-Zr-Er-Si[2], AM Al-Cu-Mn-Zr[3], Al-Ce-Mg[4] and Al-Ce-Sc-Zr-Er[5].

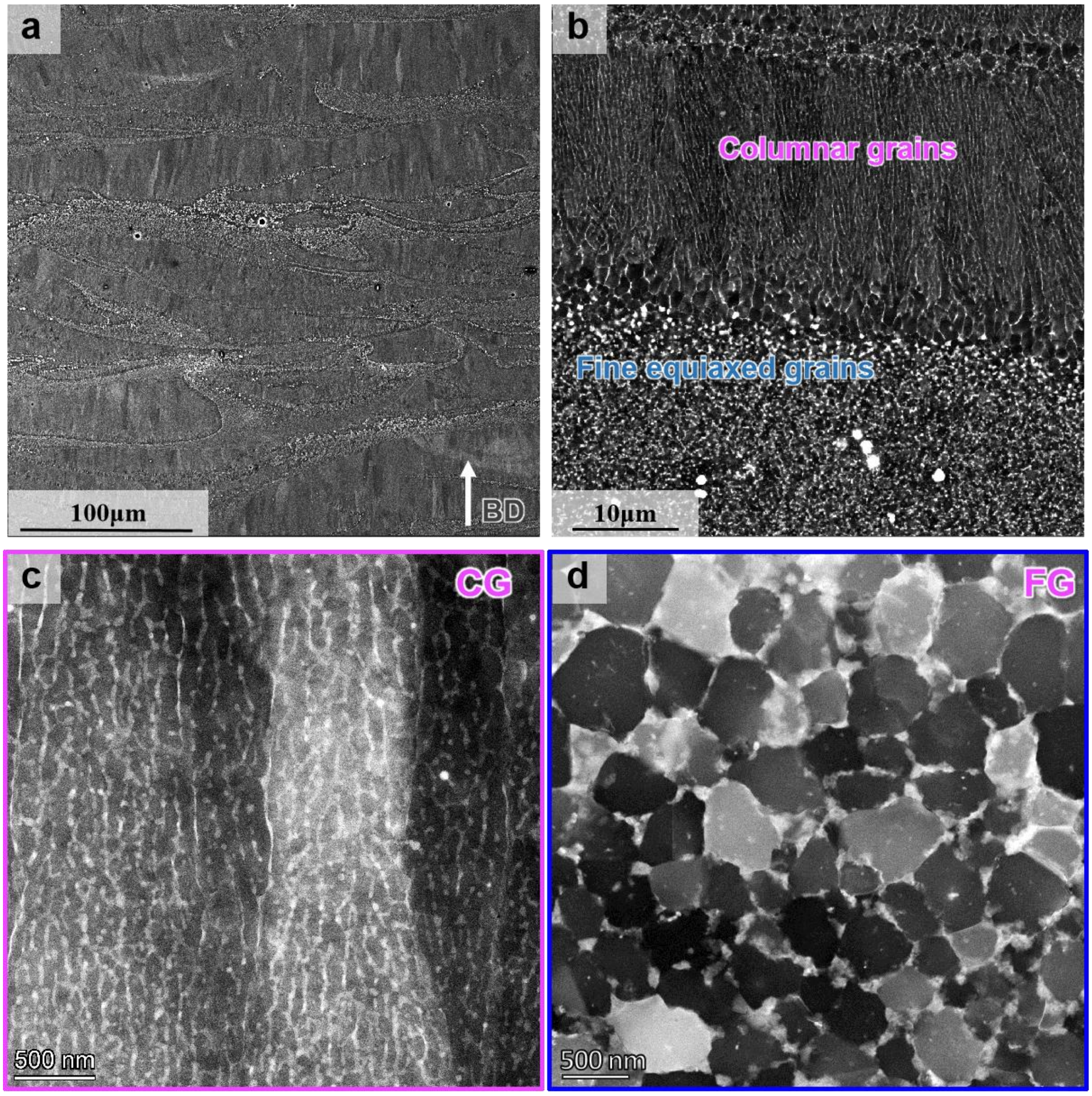

Supplementary Fig. 7 SEM and STEM observation of heat-treated IA alloy produced by PBF-LB. (a) SEM-ECCI images showing the fish scale-shaped morphology of melt pools. (b) Typical fine equiaxed grains (FG) and Columnar grains (CG) in melt pool. HAADF image of (c) Columnar grains and (d) equiaxed grains.

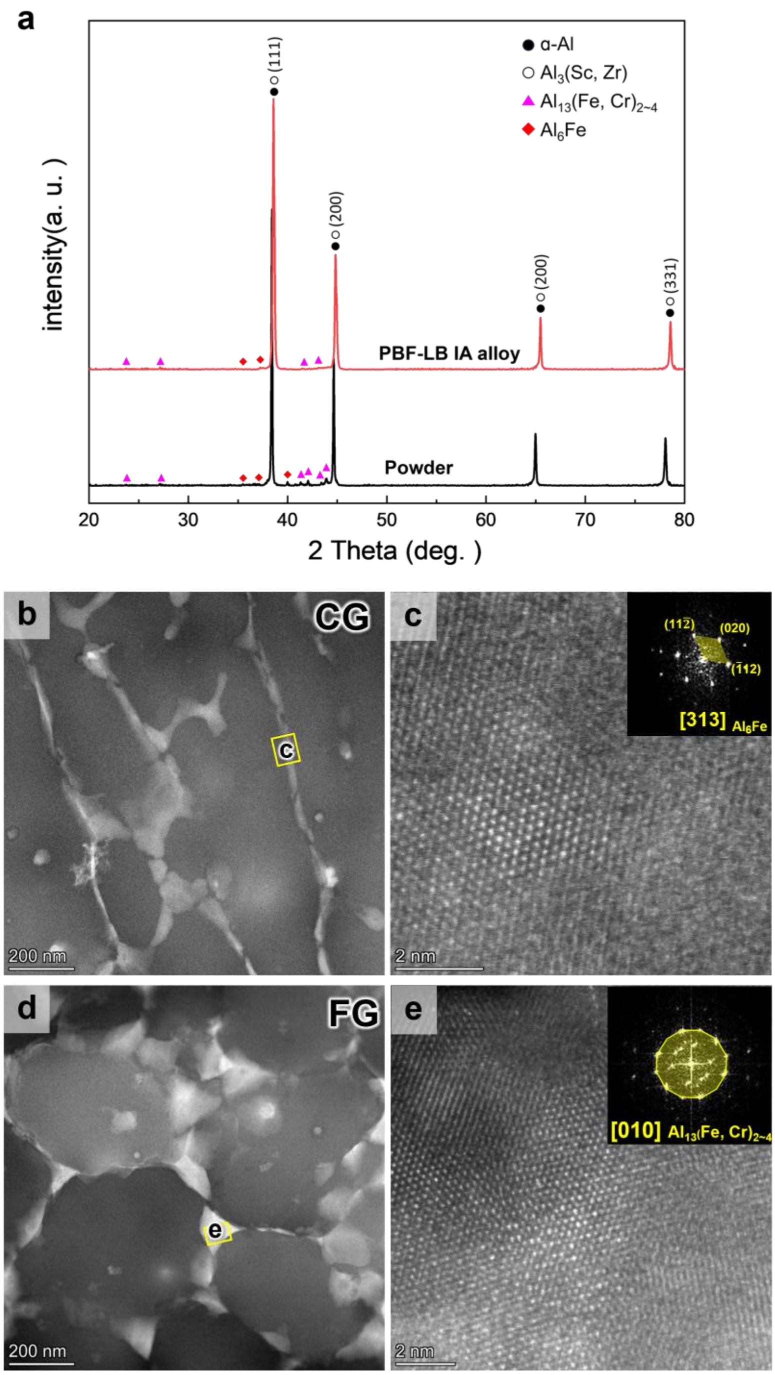

Supplementary Fig. 8 HAADF-STEM characterization of Al_6Fe and $Al_{13}(Fe, Cr)_{2\sim4}$ phase. (a) XRD spectra for powder and PBF-LB IA alloy. (b) High-angle annular dark-field (HAADF) image of the eutectic structure. (c) The High-Resolution Transmission Electron Microscopy (HRTEM) image of a selected area in (b) indicated the presence of Al_6Fe ([313]). (d) HAADF image of the $Al_{13}(Fe, Cr)_{2\sim4}$ at the grain boundaries. (e) The HRTEM image of $Al_{13}(Fe, Cr)_{2\sim4}$ with corresponding fast Fourier transform (FFT) image.

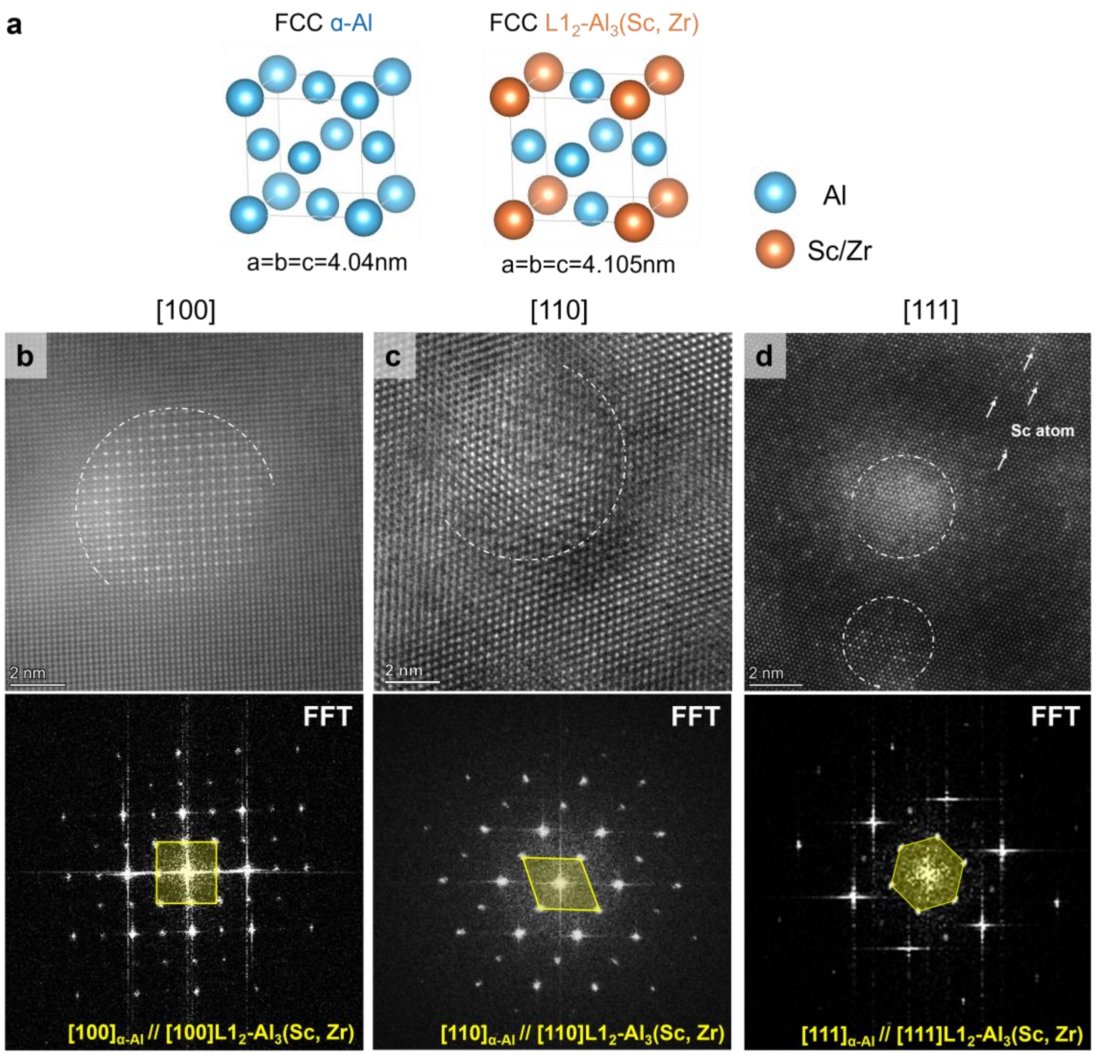

Supplementary Fig. 9 Perfect coherency between L1$_2$ precipitates and the Al matrix. (a) Lattice structures and constants of FCC Al and FCC L1$_2$-Al$_3$(Sc, Zr); (b, c, d) HRTEM images of secondary Al$_3$(Sc, Zr) particles viewed along the [110], [110] and [111] axes with corresponding FFT patterns, confirming perfect coherency between L1$_2$ precipitates and the Al matrix from different orientations.

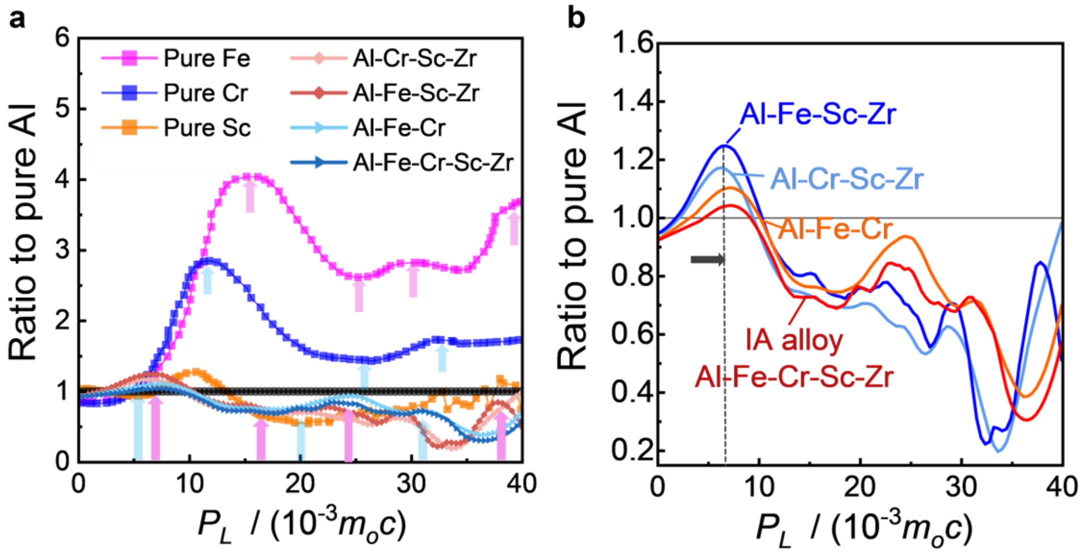

Supplementary Fig. 10 CDB ratio curves for pure Fe, pure Cr, pure Sc, AM Al-Fe-Sc-Zr, Al-Cr-Sc-Zr, Al-Fe-Cr, and IA alloys normalized to pure Al. The pink arrow corresponds to the characteristic peak of Fe, and the blue arrow corresponds to the characteristic peak of Cr. It can be observed that the characteristic peak of the IA alloy (Al-Fe-Cr-Sc-Zr alloy) is highly similar to that of pure Cr, indicating that positrons predominantly annihilate in Cr-rich regions. The error bars are standard deviations of the mean. The black arrow marks the peak in the high-momentum region. P_L is the longitudinal component of the positron-electron momentum along the direction of the γ-ray emission, c is the speed of light, m_0 is the electron rest mass.

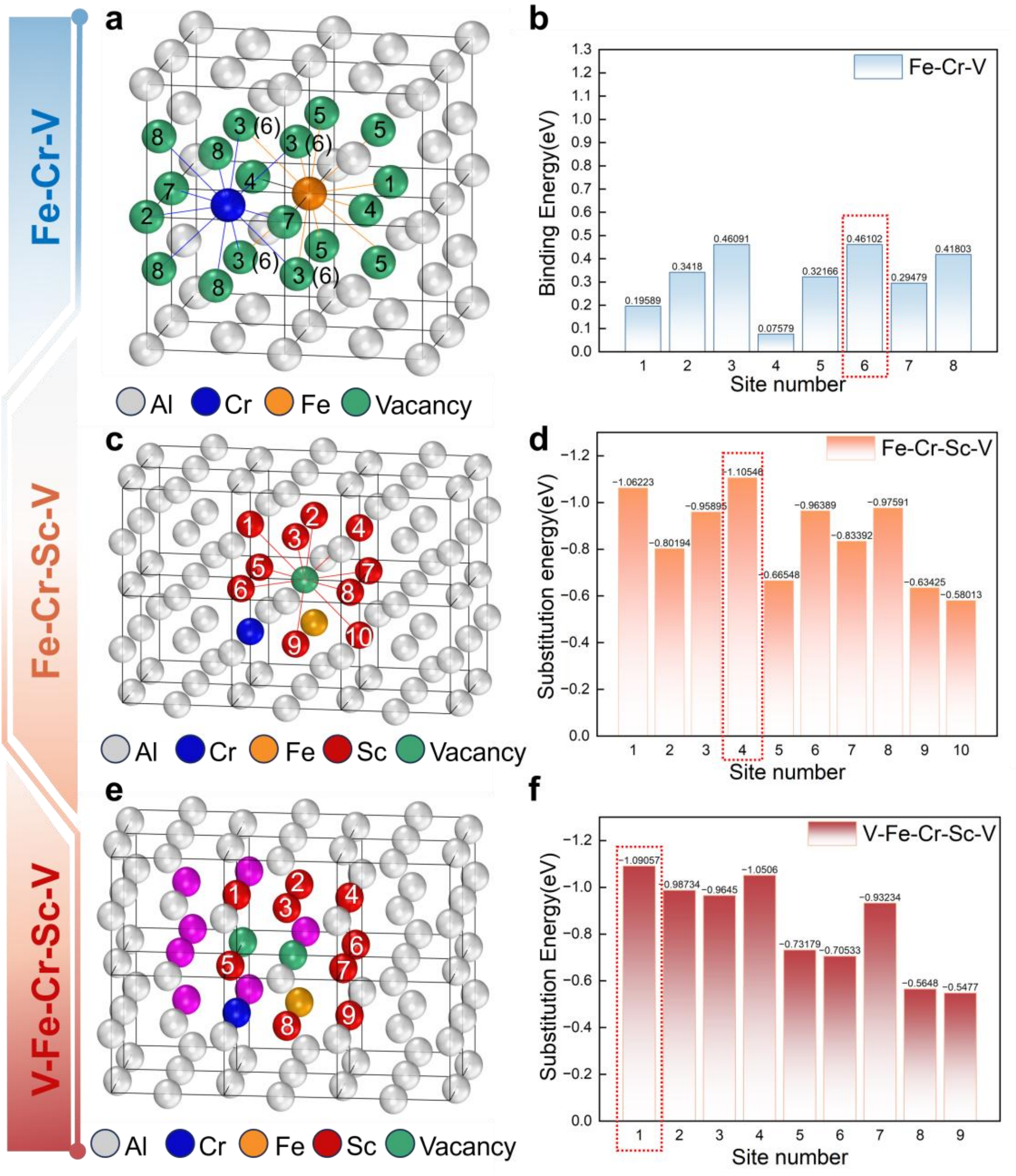

Supplementary Fig. 11 DFT calculation explores the origin of solute-vacancy clusters. (a, b) DFT-calculated binding energy for various substitution configurations of a single vacancy with Fe, Cr atom in the FCC-Al lattice. (c, d) DFT calculated the binding energies of various substitution configurations of a single Sc with Fe-Cr-V (the lowest binding energy position obtained in the previous calculation Site 3) in the FCC Al lattice. (e, f) DFT calculated the substitution energy for various substitution configurations of two Sc atoms with Fe-Cr-Sc-V (obtained in the previous calculation) in the FCC Al lattice.

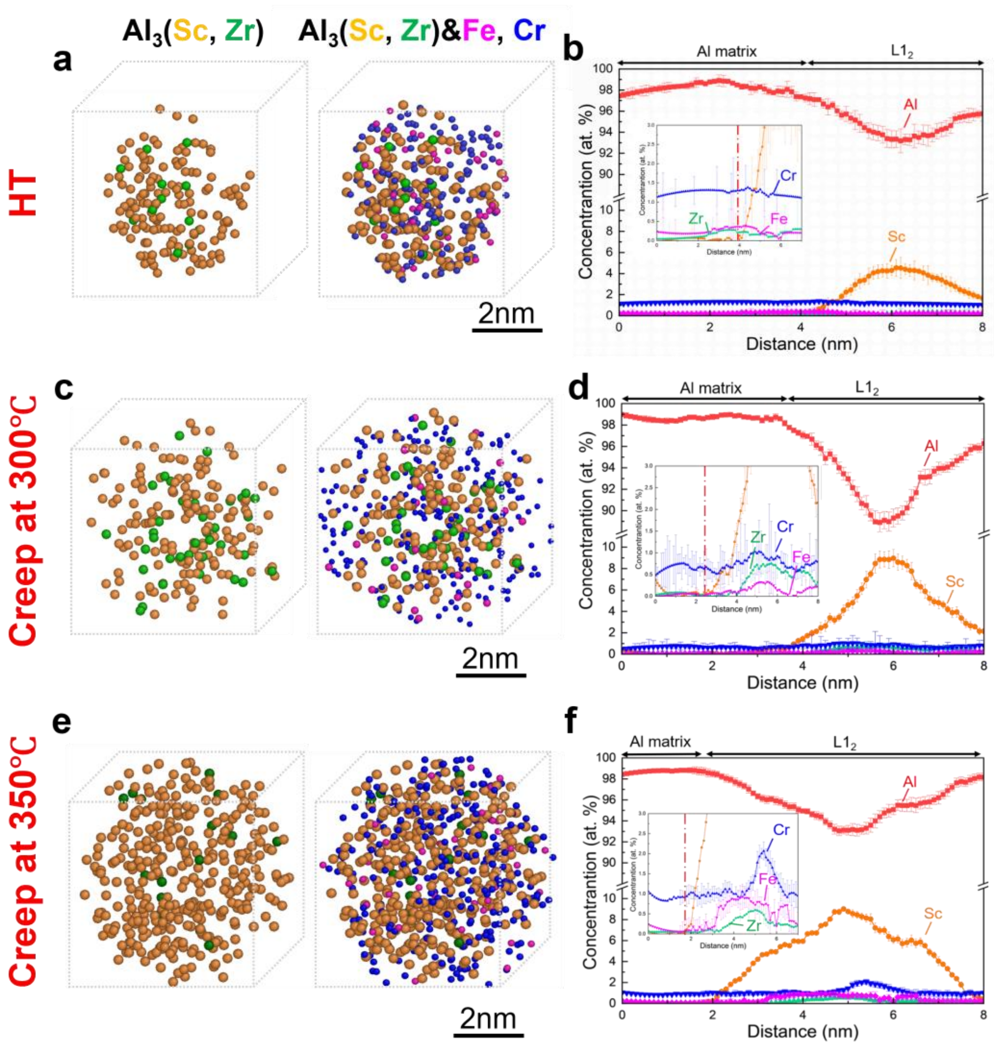

Supplementary Fig. 12 Elemental distributions in single $L1_2$ particle in IA alloy after (a) heat treating, (c) creep at 300°C and (e) creep at 350°C, with purple for Fe atoms, blue for Cr atoms, orange for Sc atoms and green for Zr atoms. (b, d, f) show concentration profiles across the matrix/L12-nanoprecipitate interface. It can be observed that with the increase of creep temperature, the Sc content inside $L1_2$ particles increases significantly, and the particle radius gradually increases. The Fe and Cr contents inside the particles also show an increasing trend.

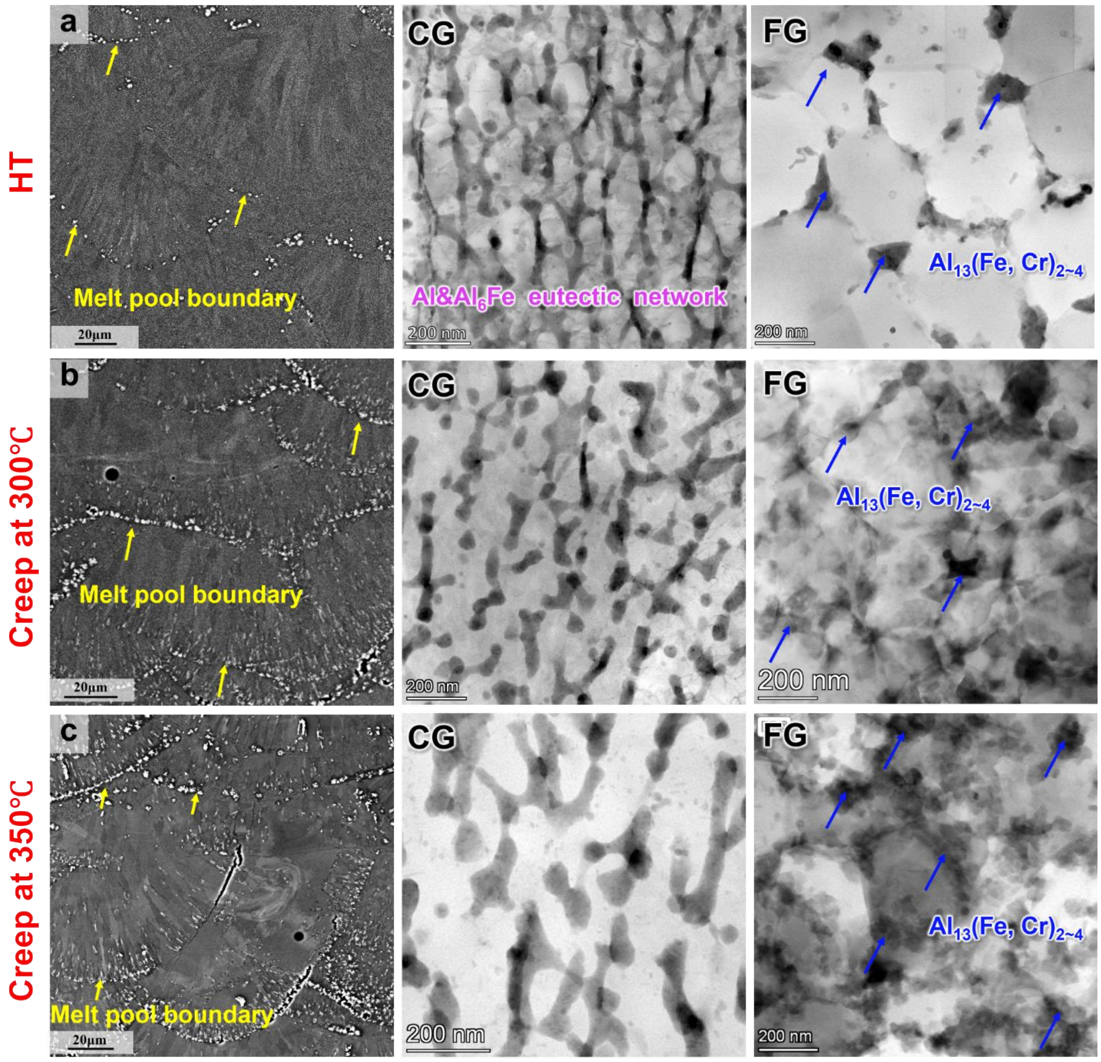

Supplementary Fig. 13 SEM and STEM images of the IA alloy after (a) heat treating, (b) creep at 300°C and (c) creep at 350°C, showing a continuous network of second phase and coarsened discontinuous dispersoids after creep at columnar grain region. $Al_{13}(Fe, Cr)_{2\sim4}$ particles located at fine equiaxed grain boundaries are pointed out by blue arrows.

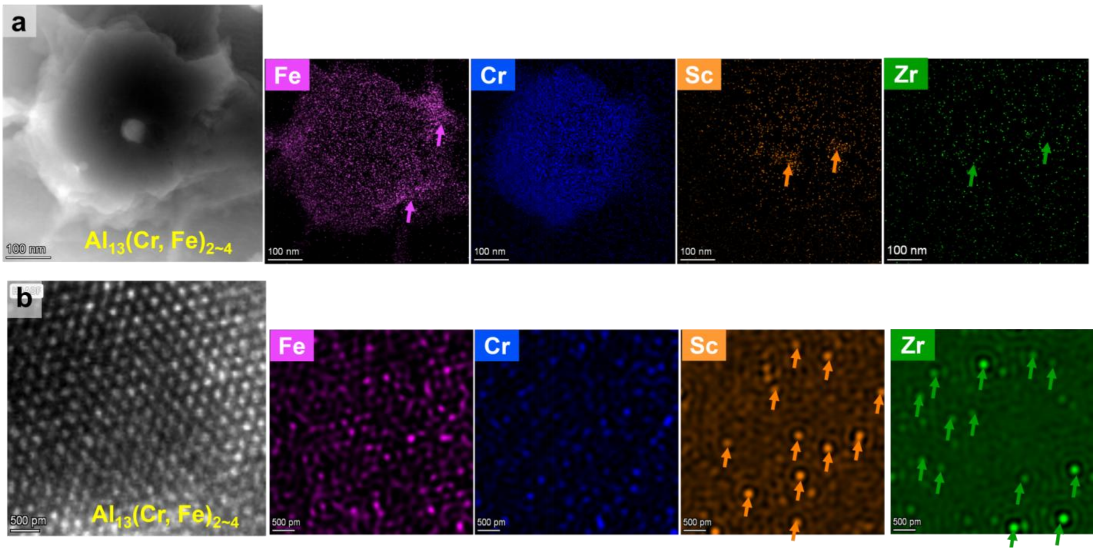

Supplementary Fig. 14 Element distribution in the $Al_{13}(Fe, Cr)_{2\sim4}$ phase after creeping. (a) TEM image and (b) High-magnification HAADF-STEM image of $Al_{13}(Fe, Cr)_{2\sim4}$ phase with corresponding EDS mapping to show the elemental distribution of Al, Fe, Cr and Sc, respectively.

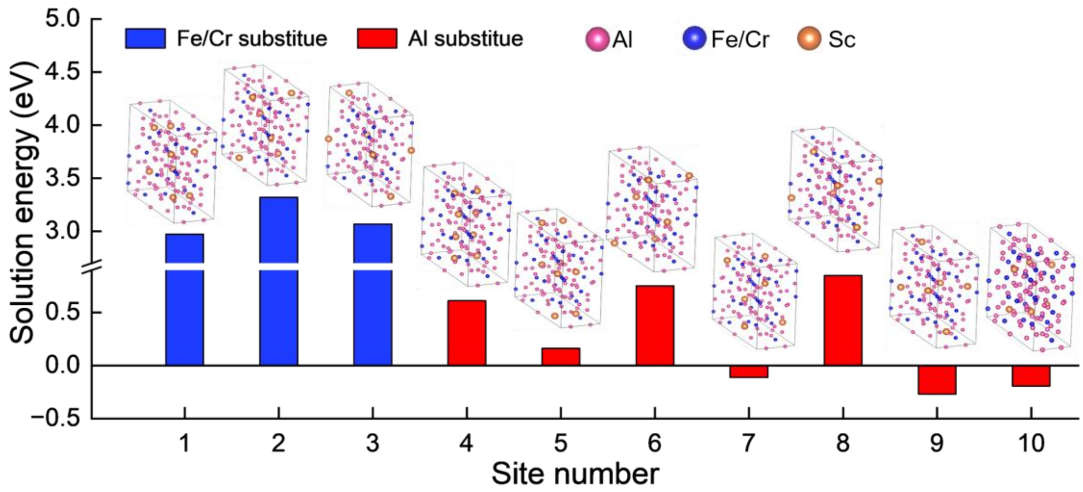

Supplementary Fig. 15 DFT-calculated substitution energy. The substitution energy for various substitution configurations of two Sc atoms within the $Al_{13}(Fe, Cr)_4$ lattice.

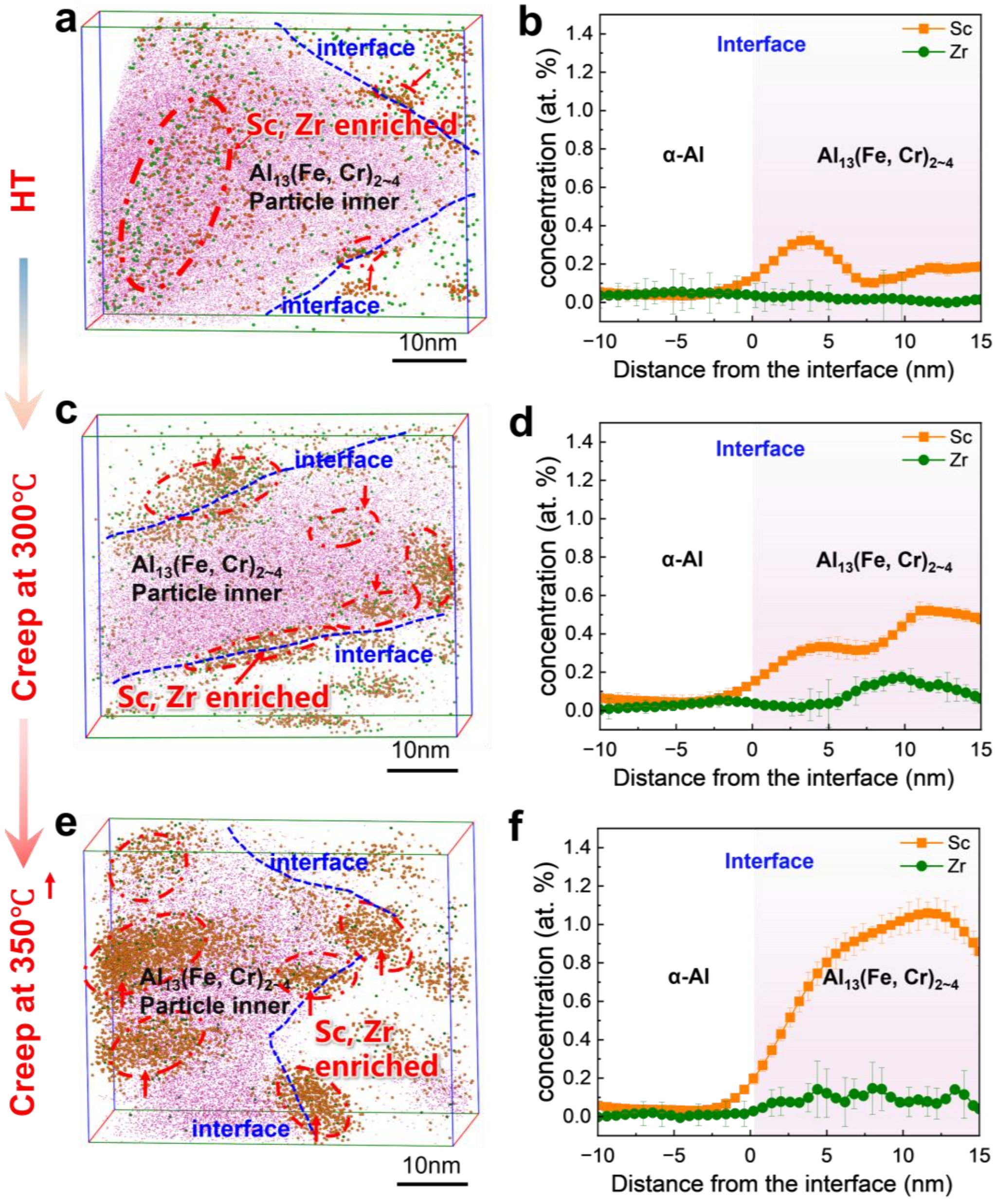

Supplementary Fig. 16 APT characterization of the $Al_{13}(Fe, Cr)_{2\sim4}$ phase. Spatial distribution of Fe, Sc and Zr atoms inside $Al_{13}(Fe, Cr)_{2\sim4}$ phase after (a) heat treating, (c) creep at 300°C and (e) creep at 350°C, with purple for Fe atoms, orange for Sc atoms and green for Zr atoms. (b)(d)(f) show the concentration of Sc/Zr inside the $Al_{13}(Fe, Cr)_{2\sim4}$ particle, where x is 0 at the interface.

Supplementary Table. 1 Chemical compositions of the IA alloy and comparative alloys

Chemical composition (wt. %)	Fe	Cr	Ni	Sc	Zr	Al
IA alloy (Al-Fe-Cr-Sc-Zr)	2.53	2.05	/	0.65	0.32	Balance
Al-Fe-Sc-Zr	4.57	/	/	0.7	0.29	Balance
Al-Cr-Sc-Zr	/	4.52	/	0.63	0.35	Balance
Al-Ni-Sc-Zr	/	/	4.54	0.61	0.31	Balance
Al-Fe-Cr	2.49	2.04	/	/	/	Balance

Supplementary Table. 2 Comparison of tensile properties for the IA alloy and comparative alloys (all after heat treatment, 325°C/4h) at various temperatures

	Heat Treatment	strengthening phases	T/°C	δ0.2/MPa	UTS/MPa	elongation/%
IA alloy (Al-2.5Fe-2Cr-Sc-Zr)	325°C 4h	$Al_{13}(Fe, Cr)_{2\sim4}$,	25	492±4	530±5	12±2
		Al_6Fe,	200	345±6	362±4	10±4
		$Al_3(Sc, Zr)$	300	275±4	305±4	7±1.5
			400	179±5	189±6	5.4±0.8
Al-4.5Fe-Sc-Zr	325°C 4h	Al_6Fe,	25	351±6	489±12	3.3±1.2
		$Al_{13}Fe_4$,	200	257±3	340±10	5.9±0.8
		$Al_3(Sc, Zr)$	300	154±7	235±5	7.6±1
			400	147±10	165±8	4.1±0.5
Al-4.5Cr-Sc-Zr	325°C 4h	$Al_{45}Cr_7$,	25	367±6	424±20	7.3±1.5
		$Al_3(Sc, Zr)$	200	247±5	294±6	8.6±0.5
			300	185±7	222±10	10±2
			400	102±4	118±12	12.9±1.5
Al-4.5Ni-Sc-Zr	325°C 4h	Al_3Ni,	25	269±8	440±12	6±2
		$Al_3(Sc, Zr)$	200	198±6	300±8	5±2
			300	116±2	134±12	4.2±1.5
			400	72±6	98±13	3±0.5
Al-2.5Fe-2Cr	325°C 4h	$Al_{13}(Fe, Cr)_{2\sim4}$,	25	334±3	387±8	10.4±1
		Al_6Fe	200	285±5	315±5	8.8±2
			300	235±4	255±6	7.2±2.3
			400	82±8	148±12	5.2±1.3

Supplementary Table. 3 Positron annihilation lifetimes, fraction of vacancy-type defects, number density of vacancies and vacancy concentration of IA alloy, Al-Fe-Sc-Zr, Al-Cr-Sc-Zr and Al-Fe-Cr alloys (all prepared by PBF-LB)

Alloys	τ_{free} (ps)	τ_{defect} (ps)	I_2/I_1	$K_{defect}(10^9 s^{-1})$	Fractions of vacancy-type defects: Vacancies associated with dislocations (*f1*) %	Monovacancies in the bulk (*f2*) %	Divacancies in the bulk (*f3*) %	Vacancy concentration ($\times 10^{-5}$ at. %)
Al-Fe-Sc-Zr	163	229	10.9	19.27	35.5	64.5	0	3.78
Al-Cr-Sc-Zr	166	231	13.023	22.07	31.1	68.8	0	4.32
Al-Fe-Cr	169	230	22.9	35.93	33.3	66.67	0	7.049
IA alloy (Al-Fe-Cr-Sc-Zr)	172	247	29.45	51.59	11.96	70.2	17.84	9.05

Note: The capture rate of positrons by defects, K_{defect}, can be estimated by:

$$K_{defect} = (\lambda_f - \lambda_d) \times I_2/I_1$$

where $\lambda_f = 1/\tau_{free}$, $\lambda_d = 1/\tau_{defect}$, I_2 and I_1 is the intensities for the second and first measured lifetimes.

Positron annihilation lifetime of defect between 220 ps and 245 ps are attributed mainly to positrons annihilating in dislocation-related vacancies (220 ps) and in bulk monovacancies (245 ps). Their relative fractions are therefore given by[6]:

$$f_1 \times 220 + f_2 \times 245 = \tau_2, \text{ with } f_1 + f_2 = 1$$

For the IA alloy, τ_2 rises to 247 ps—above the monovacancy value—indicating the additional presence of bulk divacancies (273 ps). The three defect fractions are then obtained from

$$f_1 \times 220 + f_2 \times 245 + f_3 \times 273 = \tau_2, \text{ with } f_1 + f_2 + f_3 = 1$$

where f_1 and f_2 being the fractions of dislocation-related vacancies and monovacancies and f_3 is the fraction of bulk divacancies, respectively.

Finally, the vacancy concentration is:

$$C_v = K_{defect} / \mu$$

with the specific trapping rate μ calculated as

$$\mu = f_1 \mu_{1v} + f_2 \mu_{1v} + f_3 \mu_{2v}$$

Here $\mu_{1v} = 5.097 \times 10^{14}$ s^{-1} atom^{-1} applies to both monovacancies and dislocation-related vacancies, while $\mu_{2v} = 8.481 \times 10^{14}$ s^{-1} atom^{-1} applies to bulk divacancies

Supplementary Note 1：Calculations of vacancy-atom binding energy

First-principles calculations of vacancy-atom binding energies in FCC Al were performed with the CASTEP code implemented in VASP. A 2×2×2 supercell (32Al atoms) was initially optimized using the PBEsol-GGA functional and ultrasoft pseudopotential. From this perfect lattice, three sets of defect-containing cells were constructed: (i) a single vacancy created by removing one Al atom, (ii) a single atom substituted for one Al atom at different nearest-neighbor separations from the vacancy, and (iii) a reference cell containing only the solute substitution. During the calculation, system volume, shape, and atom positions were all allowed to relax at 0 K,

The binding energy was obtained from the formula[7]:

$$-E_{binding}=E(Al_{30}\text{-}X\text{-}V)+E(Al_{32})-E(Al_{31}\text{-}X)-E(Al_{31}\text{-}V)$$

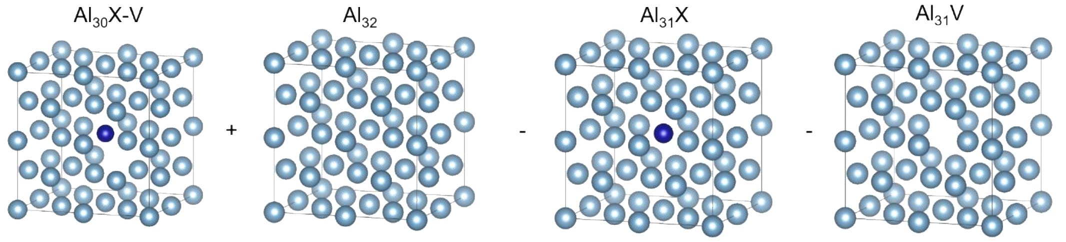

where X and V represent a solute atom and a vacancy in the Al matrix, respectively. The sign convention is adopted that positive binding energies are attractive and therefore energetically favorable.

Supplementary Note 2: Calculations of dislocation-atom interaction energy and solute redistribution around dislocation

The solute-induced stresses were calculated using the program of Vienna ab-initio Simulation Package (VASP). The generalized-gradient approximation (GGA) in the Perdew–Burke–Ernzerhof (PBE) form and the projector-augmented-wave (PAW) pseudopotentials[8]. The supercell geometry is used and the alloys are represented by $Al_{5.5}X$ supercells. The supercell translation vectors are in the same directions as the primitive translation vectors of the face-centered-cubic lattice. The energy of the $Al_{5.5}X$ supercell as a function of the lattice parameter is calculated and fitted to Birch's equation of state[9] and the lattice parameter of aluminum is determined from the minimum of the energy. The equilibrium core geometry of a pure Al *a/*2<110> edge dislocation obtained using the electronic-structure calculations and the lattice Green function flexible-boundary-condition method detailed in ref[10] served as the starting structure for the solute-dislocation interaction energies.

The enhancement of dislocation resistance is also an important part to improve the matrix strength, which can be characterized by the solute-dislocation interaction energy. The consideration in this study is focused on edge dislocations due to the pressure field it generates, which exhibits the most pronounced interaction with the solute. Due to the presence of discontinuous slip and a long-range stress field, simulating isolated dislocations is not a straightforward task. Therefore, a flexible boundary condition method proposed by Rao and Woodward was used to complete the calculation. Details are described in the Density functional theory simulations section. Outside the immediate core region, the elastic interaction energy of a dilatational solute can be formulated as:

$$U\left(x_i,y_j\right)=P\left(x_i,y_j\right)\Delta V$$

Where $P(x_i,y_j)$ is the dislocation pressure field and ΔV is the misfit volume formed by the solute atoms. $P > 0$ signifies to dilatation and $U < 0$ corresponds to binding. Calculation of ΔV is also accomplished using density functional theory. The dislocation pressure field is a function of the distance x, y between the solute atom and the slip plane:

$$P\left(x_i,y_j\right)=\frac{\mu sb(1+v)}{3\pi(1-v)}\frac{y_j}{x_i^2+y_j^2}$$

where μ is the shear modulus, b is the magnitude of the Burgers vector, s is the dislocation symbol=±1 and v is the Poisson ratio, which can be calculated using the local density approximation of DFT with a pseudopotential plane-wave method.

Solute redistribution around an edge dislocation in Al, re-drawn for Fe, Cr and Ni, while data for other elements are taken from the literature[11]. The dislocation core, located at (0, 0), is marked by a red dashed circle whose radius is approximately the magnitude of the Burgers vector; the extra half-plane extends along the Y > 0 side. Each circle delineates a region around the dislocation where the concentration of the corresponding solute species exceeds the far-field value by at least one order of magnitude. All quantum-mechanical calculations were carried out at 0 K.

Supplementary Note 3：Calculations of atom-atom binding energy

To quantify the interaction strength between two different atoms, we computed the binding energy of an A-B atomic pair from first-principles calculations, defined as:

$$-E_{binding}=E(AB)-E(A)-E(B)$$

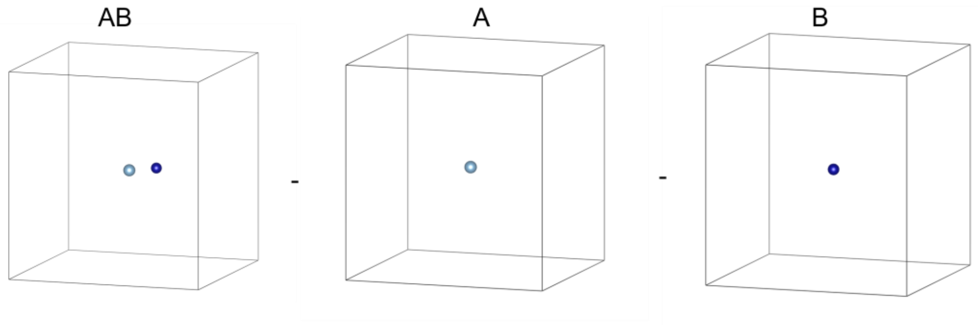

Here, *E(A)* and *E(B)* are the total energies of isolated A and B atoms, respectively, obtained by placing each atom in the center of a large vacuum supercell (18 Å × 19 Å × 20 Å) to eliminate spurious periodic interactions. The sign convention is adopted that positive binding energies are attractive and therefore energetically favorable. The combined system energy *E(AB)* was then calculated by positioning A and B at their nearest-neighbor distance (taken from experiment or literature) inside the same supercell and fully relaxing the structure. To avoid degenerate results arising from symmetry constraints, the symmetry of the supercell was intentionally lowered so that all atoms were allowed to relax without any symmetry restrictions.

Supplementary Note 4：Calculations of binding energy and substitution energy of M-V and V-M-V (M=Fe, Cr, Sc)

The binding energies between Fe, Cr and a vacancy in the Al matrix were calculated based on the DFT method[12]. A 2×2×2 conventional fcc-Al supercell (32 lattice sites) was constructed and fully relaxed under periodic boundary conditions so that residual stresses were negligible. In order to create the reference vacancy-containing cell, one Al atom was removed from the center of the relaxed supercell; the resulting vacancy was then decorated, in turn, by placing one Fe atom and one Cr atom in the nearest-neighboring (1NN) sites to the vacancy, yielding the target configuration Al_{254}(Fe-Cr-V). The binding energy $E_{binding}$ for the Fe-Cr-V cluster was then evaluated according to the thermodynamic convention

$$-E_{binding} = E(Al_{29}Fe\ Cr\ V) + E(Al_{32}) - E(Al_{30}Fe\ Cr) - E(Al_{31}V)$$

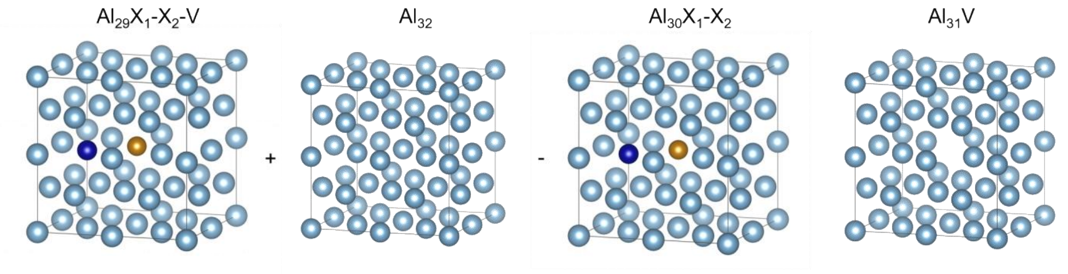

where the sign is chosen so that a positive Eb signifies an attractive, energetically favorable association; the same formula was applied mutatis mutandis to the 2NN configuration and to the isolated Fe-V and Cr-V pairs in order to decompose the total cluster energy into pairwise contributions and to quantify the synergistic stabilization provided by the simultaneous presence of both Fe and Cr around the vacancy.

Due to the difficulty in directly calculating the binding energies of triatomic single-vacancy (Fe-Cr-Sc-V) and triatomic double-vacancy (V-Fe-Cr-Sc-V) configurations caused by excessive atomic occupations, the substitution energy calculations were based on the pre-determined Fe-Cr-V configuration (identified via prior binding energy calculations). A 3×2×2 conventional fcc-Al supercell (48 lattice sites) was constructed and fully relaxed under periodic boundary conditions so that residual stresses were negligible. For the triatomic single-vacancy system, the substitution energy was evaluated when the third atom (Sc) substituted the nearest neighbor (NN) site around the vacancy in the Fe-Cr-single-vacancy structure. For the triatomic double-vacancy system, after introducing the second vacancy at the 1st nearest neighbor (1NN) site relative to the first vacancy,

the substitution energy was calculated upon substitution of the third atom (Sc) at the NN site around the vacancies. The substitution energy is defined as the energy change when an atom at the target NN site is replaced by a Sc atom, expressed as:

$$E_{sub} = E(Al_{44}\text{-}Fe\text{-}Cr\text{-}Sc\text{-}v) + E(\mu\text{-}Al) - E(Al_{45}\text{-}Fe\text{-}Cr\text{-}v) - E(\mu\text{-}Sc)$$

$$E(\mu\text{-}Al) = E(Al_{48})/48$$

$$E(\mu\text{-}Sc) = E(Al_{47}Sc) - 47E(Al)$$

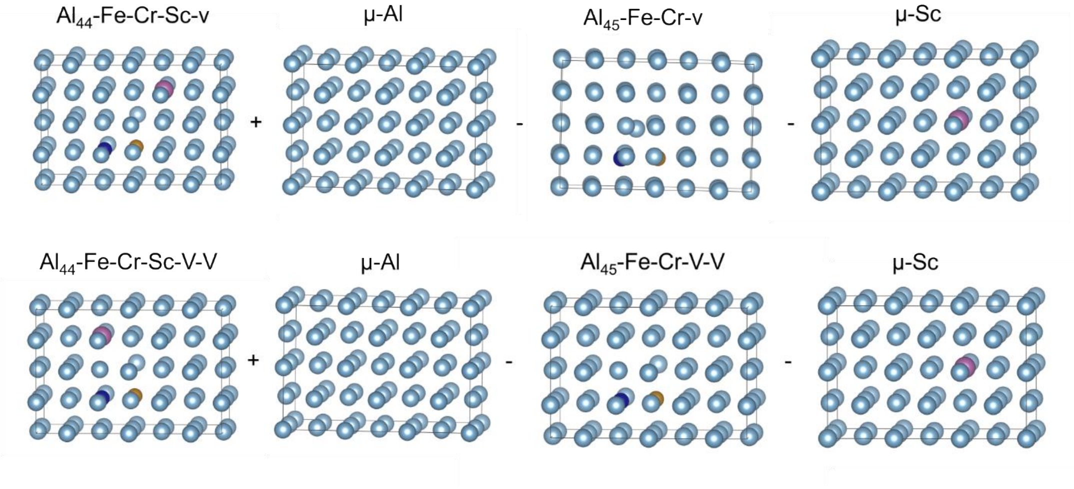

Where E (Al) refers to the energy of a single Al atom in the stable state of FCC pure Al, μ refers to potential, the supercell contains Fe sites, Cr sites, and corresponding vacancies (single or double vacancies).

Supplementary Note 5：Deformation charge density difference (DCDD) calculations

The calculation and visualization of dformation charge density were achieved through the collaboration of VASP and VESTA. DCDD represents the charge density difference between the self-consistently converged system and the superposition of spherically symmetric charge densities of individual constituent atoms in their free states. The calculation formula is:

$$\Delta=\rho(AB_{\text{self-consistent}})-\rho(AB_{\text{atomic}})$$

The resulting CHGCAR files were imported into VESTA 3.5.7 and processed on a common FFT grid. Iso-surface maps were rendered at ±0.04 e $bohr^{-3}$ (blue= depletion, red= accumulation) to visualize charge transfer and bonding perturbations around the vacancy.

Supplementary Note 6: Calculations of substitution energy

The substitution energy defined as the energy change when an atom of $Al_{13}(Fe, Cr)_4$ is replaced by a Sc atom, is written as[13]:

$$E_{Sc\rightarrow Al} = [E(Al_{13N-1}Fe/Cr_{4N}Sc) + E(Al)] - [E(Al_{13N}(Fe, Cr)_{4N}) + E(Sc)]$$

$$E_{Sc\rightarrow Fe/Cr} = [E(Al_{13N}\,Fe/Cr_{4N-1}Sc) + E(Fe/Cr)] - [E(Al_{13N}(Fe, Cr)_{4N}) + E(Sc)]$$

where the super-cell is considered to contain 13 N Al sites and 4 N Fe/Cr sites, E is the ground state energy of a super-cell with atom composition given in parenthesis, E(Al), E(Fe/Cr) and E(Sc) are the energy per atom of the corresponding bulk element assuming same cell symmetry. The relative energy is the normalized total energy for a given distribution configuration with two Sc atoms, i.e., $E_{relative} = E_{total}\text{-}max(E)$, where Etotal is the total system energy and max(E) is maximum total energy obtained in the calculated configurations. The calculations employed a single 102-atom conventional cell of $Al_{13}Fe_4$ whose initial lattice parameters (a = 15.489 Å, b = 8.083 Å, c = 12.496 Å, β = 107.75°) were taken from experiment and fully relaxed under GGA-PBE; the same cell was then duplicated to generate a 1 × 1 × 1 supercell in which one Al atom occupying the 8j Wyckoff position was selected for replacement by Sc, ensuring negligible elastic interaction between periodic images. Geometry optimizations were carried out with ultrasoft pseudopotentials (valence configurations Al $3s^23p^1$, Fe $3d^64s^2$, Sc $3d^14s^2$), a plane-wave cutoff of 450 eV, and a Monkhorst–Pack 4 × 4 × 2 k-mesh, with all atomic positions, cell shape, and volume simultaneously relaxed until energy changes fell below 5×10^{-6} eV atom^{-1}, residual forces below 0.01 eV Å^{-1}, stresses below 0.02 GPa, and displacements below 5×10^{-4} Å; spin polarization was included with an initial ferromagnetic ordering on Fe sublattices. The total energies of the pristine $Al_{13}Fe_4$ supercell, the Sc-substituted $Al_{12}Sc(Fe, Cr)_4$ supercell, and the reference elemental phases were extracted from fully relaxed calculations under identical computational settings.

Supplementary Note 7：Positron annihilation

Positron annihilation lifetime spectroscopy (PALS) is a highly sensitive technique for detecting open-volume defects in crystalline materials, including vacancies, vacancy clusters and dislocations[6]. When positrons are injected into a solid, they rapidly thermalize and diffuse through the lattice before annihilating with electrons. In defect-free regions, positrons annihilate with relatively high electron density and therefore exhibit shorter lifetimes. In contrast, positrons can become trapped at open-volume defects where the local electron density is reduced, leading to longer positron lifetimes. As a result, the positron lifetime spectrum typically consists of multiple lifetime components corresponding to annihilation in the bulk lattice and at different types of defects. In this work, PALS was used to characterize vacancy-type defects and their relative concentrations in additively manufactured Al alloys. The measured lifetime spectra were decomposed into several exponential components, where each lifetime component (τi) corresponds to a specific annihilation state and the associated intensity (Ii) reflects the relative fraction of positrons annihilating in that state. The average positron lifetime can therefore provide information about the overall defect density in the material.

Coincidence Doppler broadening spectroscopy (CDBS) is an advanced positron annihilation technique used to probe the chemical environment surrounding positron annihilation sites[14]. When a positron annihilates with an electron in a solid, two γ photons with an energy close to 511 keV are emitted in opposite directions. Because electrons in the material possess finite momentum, the energy of the emitted γ photons deviates slightly from 511 keV. This Doppler broadening of the annihilation peak reflects the momentum distribution of the electron–positron pair. Core electrons possess significantly higher momentum than valence electrons, and their momentum distribution is strongly dependent on the atomic species. Therefore, analysis of the high-momentum region of the Doppler-broadened spectrum provides information about the elemental environment around the annihilation site. In defect studies, this makes CDBS particularly useful for identifying the types of atoms surrounding vacancies or vacancy clusters where positrons are preferentially trapped.

Supplementary References:

1 Farkoosh, A. R., Dunand, D. C. & Seidman, D. N. Enhanced age-hardening response and creep resistance of an Al-0.5Mn-0.3Si (at.%) alloy by Sn inoculation. *Acta Materialia* **240**, 118344 (2022). https://doi.org/https://doi.org/10.1016/j.actamat.2022.118344

2 De Luca, A., Seidman, D. N. & Dunand, D. C. Mn and Mo additions to a dilute Al-Zr-Sc-Er-Si-based alloy to improve creep resistance through solid-solution- and precipitation-strengthening. *Acta Materialia* **194**, 60-67 (2020). https://doi.org/https://doi.org/10.1016/j.actamat.2020.04.022

3 Michi, R. A. *et al.* Load shuffling during creep deformation of an additively manufactured AlCuMnZr alloy. *Acta Materialia* **244**, 118557 (2023). https://doi.org/https://doi.org/10.1016/j.actamat.2022.118557

4 Ng, D. S. & Dunand, D. C. Aging- and creep-resistance of a cast hypoeutectic Al-6.9Ce-9.3Mg (wt.%) alloy. *Materials Science and Engineering: A* **786**, 139398 (2020). https://doi.org/https://doi.org/10.1016/j.msea.2020.139398

5 Ekaputra, C. N., Rakhmonov, J. U., Weiss, D., Mogonye, J.-E. & Dunand, D. C. Microstructure and mechanical properties of cast Al-Ce-Sc-Zr-(Er) alloys strengthened by Al11Ce3 micro-platelets and L12 Al3(Sc,Zr,Er) nano-precipitates. *Acta Materialia* **240**, 118354 (2022). https://doi.org/https://doi.org/10.1016/j.actamat.2022.118354

6 Su, L. H. *et al.* Study of vacancy-type defects by positron annihilation in ultrafine-grained aluminum severely deformed at room and cryogenic temperatures. *Acta Materialia* **60**, 4218-4228 (2012). https://doi.org/https://doi.org/10.1016/j.actamat.2012.04.003

7 Wolverton, C. Solute–vacancy binding in aluminum. *Acta Materialia* **55**, 5867-5872 (2007). https://doi.org/https://doi.org/10.1016/j.actamat.2007.06.039

8 Kresse, G. & Furthmüller, J. Efficient iterative schemes for ab initio total-energy calculations using a plane-wave basis set. *Physical Review B* **54**, 11169-11186 (1996). https://doi.org/10.1103/PhysRevB.54.11169

9 Birch, F. Finite strain isotherm and velocities for single-crystal and polycrystalline NaCl at high pressures and 300°K. *Journal of Geophysical Research: Solid Earth* **83**, 1257-1268 (1978). https://doi.org/https://doi.org/10.1029/JB083iB03p01257

10 Leyson, G. P. M., Curtin, W. A., Hector, L. G. & Woodward, C. F. Quantitative prediction of solute strengthening in aluminium alloys. *Nature Materials* **9**, 750-755 (2010). https://doi.org/10.1038/nmat2813

11 Vannarat, S., Sluiter, M. H. F. & Kawazoe, Y. First-principles study of solute-dislocation interaction in aluminum-rich alloys. *Physical Review B* **64**, 224203 (2001). https://doi.org/10.1103/PhysRevB.64.224203

12 Peng, J., Bahl, S., Shyam, A., Haynes, J. A. & Shin, D. Solute-vacancy clustering in aluminum. *Acta Materialia* **196**, 747-758 (2020). https://doi.org/https://doi.org/10.1016/j.actamat.2020.06.062

13 Yi, M. *et al.* Atomic-scale compositional complexity ductilizes eutectic phase towards creep-resistant Al-Ce alloys with improved fracture toughness. *Acta Materialia* **276**, 120133 (2024). https://doi.org/https://doi.org/10.1016/j.actamat.2024.120133

14 Lab, A. N., Scientific, U. S. D. o. E. O. o. & Information, T. *Positron-annihilation Spectroscopy of Defects in Metals: An Assessment*. (Argonne National Lab, 1982).
